# An urgent call to address work-related psychosocial hazards and improve worker well-being

**DOI:** 10.1002/ajim.23583

**Published:** 2024-04-10

**Authors:** Paul A. Schulte, Steven L. Sauter, Sudha P. Pandalai, Hope M. Tiesman, Lewis C. Chosewood, Thomas R. Cunningham, Steven J. Wurzelbacher, Rene Pana-Cryan, Naomi G. Swanson, Chia-Chia Chang, Jeannie A. S. Nigam, Dori B. Reissman, Tapas K. Ray, John Howard

**Affiliations:** 1 Advanced Technologies and Laboratories International Inc., Gaithersburg, Maryland, USA; 2 Division of Science Integration, National Institute for Occupational Safety and Health, Centers for Disease Control and Prevention, Cincinnati, Ohio, USA; 3 Division of Safety Research, National Institute for Occupational Safety and Health, Centers for Disease Control and Prevention, Morgantown, West Virginia, USA; 4 Office of the Director, National Institute for Occupational Safety and Health, Centers for Disease Control and Prevention, Atlanta, GA, USA; 5 Division of Field Studies and Engineering, National Institute for Occupational Safety and Health, Centers for Disease Control and Prevention, Cincinnati, OH, USA; 6 Office of the Director, National Institute for Occupational Safety and Health, Centers for Disease Control and Prevention, Washington, DC, USA; 7 Office of the Director, National Institute for Occupational Safety and Health, Centers for Disease Control and Prevention, Cincinnati, OH, USA

**Keywords:** economics, mental health, occupational safety and health, psychological effects, work organization

## Abstract

Work-related psychosocial hazards are on the verge of surpassing many other occupational hazards in their contribution to ill-health, injury, disability, direct and indirect costs, and impact on business and national productivity. The risks associated with exposure to psychosocial hazards at work are compounded by the increasing background prevalence of mental health disorders in the working-age population. The extensive and cumulative impacts of these exposures represent an alarming public health problem that merits immediate, increased attention. In this paper, we review the linkage between work-related psychosocial hazards and adverse effects, their economic burden, and interventions to prevent and control these hazards. We identify six crucial societal actions: (1) increase awareness of this critical issue through a comprehensive public campaign; (2) increase etiologic, intervention, and implementation research; (3) initiate or augment surveillance efforts; (4) increase translation of research findings into guidance for employers and workers; (5) increase the number and diversity of professionals skilled in preventing and addressing psychosocial hazards; and (6) develop a national regulatory or consensus standard to prevent and control work-related psychosocial hazards.

## 1 | INTRODUCTION

Exposure to work-related psychosocial hazards is projected to become a major occupational health and safety threat, with significant implications for workers, businesses, and the national economy.^1–9^ This threat may affect many of the 169.6 million US workers by 2030 and result in adverse mental and physical health, leading to increased morbidity, mortality, and disability.^10,11^ In turn, these effects could have major impacts on national, business, and worker economic circumstances.^12^ Given the growing evidence of the connections between work and health outcomes, there is a pressing need to prevent work-related psychosocial hazards and the adverse cognitive, emotional, behavioral, physiological, and economic effects associated with them.^6,12–14^ This paper is a commentary that describes the critical national problem of exposure to psychosocial hazards and resultant adverse effects. The paper provides a narrative and nongraded summary of the scientific literature and identifies six societal actions that can help address the problem of work-related psychosocial hazards.

Work-related psychosocial hazards are aspects of the design and management of work and its social-organizational context that have the potential to cause physical and psychological harm (Table 1).^79^ Beyond their effects on health, psychosocial hazards can impair workers’ ability to participate effectively in the work environment and with other people in and outside of work.^80^ Table 2 presents a summary of the behavioral, mental, and physical health effects reported as being associated with work-related psychosocial hazards.

Exposure to work-related psychosocial hazards is widespread, and, in *Mental health at work: a review of interventions in organizations*, Silvaggi and Miraglia note that “the workplace can negatively affect workers’ mental health by intensifying an existing situation or contributing to the development of mental health conditions via exposure to excessive work stressors.”^81^ The relationship between work and mental health is also bidirectional, where mental and physical health can influence work performance.^82^ The complexity of psychosocial hazards and mental health associations present significant challenges for understanding these relationships and addressing such hazards to mitigate the burden and stigma of mental health outcomes in working populations.

**FIGURE 1:**
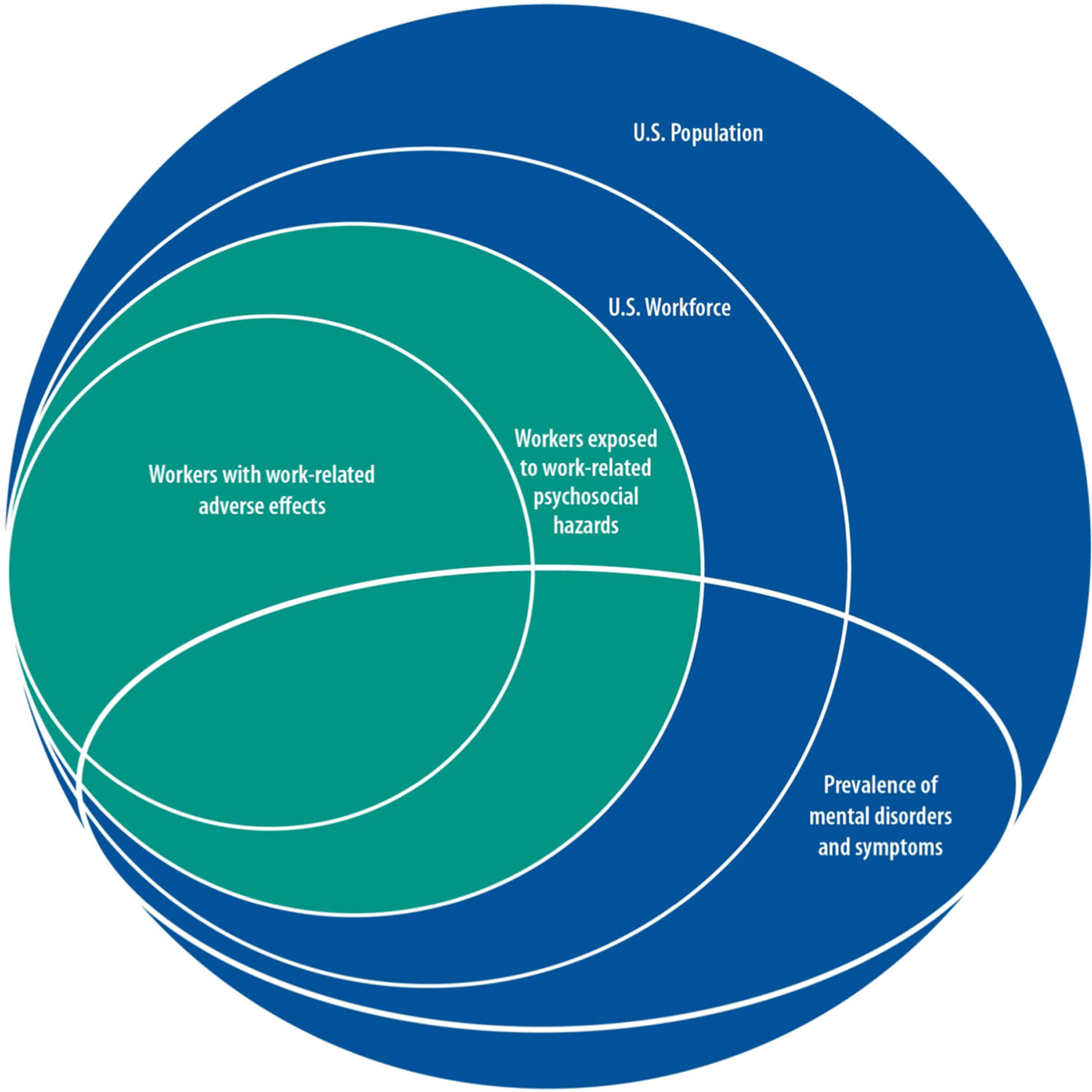
Conceptual map of the US burden of adverse effects from work-related psychosocial hazards.

Concerns over work-related psychosocial hazards are compounded by the increasing prevalence of mental health disorders in the population.^12^ Seventy-six percent of workers reported at least one symptom of a mental health condition, which increased by 17% in just 2 years.^83^ Figure 1 conceptualizes a nested set of domains beginning with the US general population, proceeding to the US workforce, and progressing to those workers with work-related psychosocial exposures. The smallest domain includes workers with adverse health effects from work-related exposures. The intersection of the prevalence of mental disorders across the nested population domains highlights that some portion of adverse worker health effects are mental health in nature and that some baseline of mental health conditions in a worker that might be observed in the general population could be relevant for health effects associated with exposure to psychosocial hazards.

In the USA, for many reasons, the time is right to address psychosocial hazards more aggressively. First, the prevalence and impact of psychosocial hazards in today’s workplaces appear to be escalating.^13,49,84^ Second, the changing nature of work due to non-standard work arrangements and resultant precariousness of work underpins the increase of adverse health effects.^85–87^ Third, the COVID-19 pandemic increased awareness that work is a social determinant of health and that work-related hazards can have a major impact on mental health.^88–92^ Fourth, the scientific and public health communities are calling for an expanded focus for occupational safety and health (OSH) to address psychosocial hazards and well-being.^3,9,80,83,93–96^ Fifth, the National Institute for Occupational Safety and Health (NIOSH) has established a foundational approach, *Total Worker Health*^®^, focusing on the design and organization of work and nonwork factors that affect the well-being of workers.^97^ This approach is a holistic perspective that focuses on how work affects overall health and well-being, including physical, psychological, social, and economic aspects.^98,99^ Sixth, there are efforts to achieve parity between mental and physical health in workers’ compensation insurance coverage so that the former is no longer treated as a “second-tier” health condition.^100^ Finally, many countries and international organizations have developed policies on psychosocial hazards,^1,6,9,17,101–103^ though the USA has not. For further information on mental health and well-being in the workplace, see Supporting Information S1: SI 1.

## 2 | BASIS FOR ACTION

In this paper, we call for action based on three questions: (1) How strong is the link between exposure to work-related psychosocial hazards and adverse effects on workers? (2) How large is the health and economic burden of these hazards and effects? (3) What can employers do to address work-related psychosocial hazards? To answer these questions, we draw upon national and international research, authoritative policies and frameworks, and NIOSH guidance on reducing work-related psychosocial hazards.^1,6,11,80,89,104,105^ In the following sections, we present our findings and conclude with a call to action which outlines six actions that may reduce psychosocial hazards at work and improve worker mental health and well-being. We begin by discussing how work affects well-being.

## 3 | THE LINK BETWEEN WORK-RELATED PSYCHOSOCIAL HAZARDS AND ADVERSE HEALTH EFFECTS

Harvey et al. (2017) conducted a systematic meta-review of the literature on work and mental health conditions (depression, anxiety, and stress-related conditions).^22^ The review found a moderate level of evidence for associations with health effects for work organization variables, including high job demand, low job control, low workplace social support, effort-reward-imbalance, low organizational procedural justice, low organizational relational justice, organizational change, job insecurity, temporary employment status, atypical working hours, bullying, and role stress.^22^ In a more recent meta-analysis, Niedhammer et al. (2021) also found significant associations between job strain, effort-reward imbalance, job insecurity, and long working hours with coronary heart disease (CHD), stroke, and depression.^11^

One of the work-related hazards with a significant body of research is job strain. Job strain results from exposure to job stressors such as the combination of work with high demands and low control. Meta-analyses have found job strain to be associated with a 23% increase in CHD^28^ and a 30% increase in the risk of stroke.^106^ Another systematic review of papers published between 1985 and 2014 found workers who reported job stressors, including job strain, had an increased incidence of ischemic heart disease.^107^ In a meta-analysis of European cohort studies Kivimäki and colleagues (2012) found, after adjustment for sex and age, a hazard ratio of 1.23 (95% CI: 1.10, 1.37) for CHD among those reporting job strain.^27^

Workplace violence (WPV) is another psychosocial hazard that has been shown to negatively impact workers’ physical and mental health. A recent systematic review of 24 studies found associations between WPV and poor mental health and psychological distress.^65^ The effects of WPV have been relatively well-studied, particularly among healthcare providers. WPV is associated with adverse mental health, depression, anxiety, posttraumatic stress disorder (PTSD), burnout, sleep problems, increased use of antidepressants, and decreased job satisfaction and quality of life.^65^

Psychosocial hazards are also associated with chronic and traumatic injuries. A 2021 article identified 24 systematic reviews and 5 longitudinal studies and found evidence of generally consistent findings for an association of job demands, job strain, effort/reward-imbalance, and increased risk for workplace musculoskeletal disorders, though the authors concluded there was insufficient evidence linking psychosocial hazards with an increased risk for traumatic injuries.^108^ However, other longitudinal studies have reported significant associations between psychosocial hazards and increased risk for injury, especially among certain sub-populations, such as older workers.^109,110^

The workplace psychosocial environment can influence worker well-being along multiple pathways. Most commonly cited are psychophysiological effects of stress, which result from a chronic imbalance between work demands and ability to cope with those demands^111,112^; from efforts to conserve resources^113^; and from imbalance of effort and rewards.^114^ Other cited research involves the relationship between allostatic load—the physiological measure of cumulative stress on the body leading to cardiovascular diseases— and other health conditions.^115^ Sorensen et al. (2016) illustrated in a conceptual model that work-related psychosocial factors may also influence health and safety behaviors and engagement in workplace health programs, and in turn, influence health and safety outcomes as well as enterprise outcomes (such as absences and turnover).^116^

While the case that work-related psychosocial hazards are causal factors for adverse health effects is strongly supported in the literature,^11,117–119^ there remains some concern over causality. Critics argue that many of the studies are cross-sectional and only describe associations. Also, conclusions are limited because of the use of self-reported data. Although more recently, prospective studies have been conducted on outcomes such as cardiovascular disease and depression, self-reporting is still an issue.^40,120^ However, causal inference always involves some level of judgment based on integrating diverse types of evidence.^121^ When this is done, the collective body of literature on work-related psychosocial factors suggests that controlling psychosocial hazards will prevent or reduce adverse physiological and psychological outcomes such as those shown in Table 2.^11,17,87,91,122–124^ We next examine the substantial burden and costs associated with work-related psychosocial hazards.

## 4 | BURDEN OF WORK-RELATED PSYCHOSOCIAL HAZARDS AND ADVERSE PHYSICAL AND MENTAL HEALTH EFFECTS

### 4.1 | Exposure to work-related psychosocial hazards

Most workers have the potential to be exposed to some degree of work-related psychosocial hazards due to meeting expectations and deadlines, working or interacting with others, balancing work with life responsibilities, and coping with difficult work processes.^6,9,117^ Table 3 displays the estimated national prevalence of psychological hazards in 2018. Close to 30% of workers responded that they, either always or often, found their work stressful. Almost 70% agreed that they had to work very fast, and 43% perceived that demand at their job interferes with their family life. Approximately a quarter of workers believed they do not have any decision-making power at work, and a similar percentage reported an inability to take time off work when needed. Another psychosocial hazard not included in the table is WPV. WPV appears to be increasing. Between 2015 and 2019, nonfatal WPV events among workers increased by 25%.^125^ WPV can result in physical, psychological, and financial costs to workers and their employers. See Supporting Information S1: SI 2 for further information on exposure to work-related psychosocial hazards.

### 4.2 | Prevalence of adverse mental health effects

Depressive and anxiety disorders are among the leading causes of disability burden worldwide. Prevalence estimates and resulting disability are higher than most chronic diseases.^126^ More than 47% of Americans are expected to be diagnosed with a mental health disorder at some point in their lifetime.^127^ Therefore, it is likely that most workplace managers, employers, and workers will engage with a coworker with a mental health condition at work. Overall, 2.7% of working adults experienced some form of serious psychological distress.^128^ Daly, using data from the National Health Interview Survey, concluded there has been an upward trend in reported psychological distress among working people in the United States, increasing 40% over the period 1999–2018.^129^ See Supporting Information S1: SI 3 for background information on mental health disorders.

### 4.3 | Economic burden of work-related psychosocial hazards

Goh et al. (2016) assessed direct US medical costs of exposure to 10 work-related psychosocial hazards at $187 billion (in 2014 dollars).^130^ These stressors included unemployment, lack of health insurance, exposure to shift work, long working hours, job insecurity, work-family conflict, low job control, high job demand, low social support at work, and low organizational justice.^130^ The total cost, including indirect and intangible costs from these exposures, was not assessed, but if it had, it would likely be much higher.^131^ Exposure to work-related psychosocial hazards also results in the decline of non-pecuniary economic outcomes such as workplace productivity and workers’ health-related quality of life. As one example, a study found that workers exposed to precarious working conditions on average lose 0.4 healthy days and have 1.2 days of limited activity within a 30-day period.^49^ Further information on the economic burden of psychosocial hazards may be found in Supporting Information S1: SI 4.

### 4.4 | Assessing the effects of psychosocial hazards in workers’ compensation claims

Workers’ compensation (WC) systems provide limited but useful information on the adverse effects of psychosocial hazards, but considerations around compensability related to mental health conditions vary from state to state. Mental health conditions may appear in WC systems in three main ways.^132^ One is physical–mental, where a physical injury/illness leads to or exacerbates a mental health condition. Another is mental–physical, where a mental stimulus (psychological stressor) leads to or exacerbates a physical condition. A third is mental–mental, where a mental stimulus exacerbates a mental health condition.

The physical–mental type comprises most mental health WC claims^132^ since all states allow these types of claims. However, the proportion of physical–mental WC cases is difficult to pinpoint, since these cases can only be identified through detailed claims review for mental health diagnoses, treatments, and medications. Based on 2015–2017 private sector WC data from California, mental stress and mental disorder claims (where the primary coded nature of injury was mental-related) represented 1.3% of all claims.^95^ By contrast, based on 2014–2016 private sector WC data from Tennessee, mental stress and mental disorder claims represented only 0.09% of all claims.^133^ Although the frequency of mental–physical or mental–mental WC claims is low, it is increasing in US states.^95,132^ This may be due to an increase in state WC laws to cover these claims.^132,134^ COVID-19 has also increased the number of first responders, healthcare providers, and others reporting mental-related claims, and there may be an increased awareness of mental health conditions among employers and workers.^132,134^ The cost for claims is discussed in Supporting Information S1: SI 5.

## 5 | WHAT CAN EMPLOYERS DO TO ADDRESS WORK-RELATED PSYCHOSOCIAL HAZARDS?

### 5.1 | The hierarchy of controls and work-related psychosocial hazards

In OSH, the hierarchy of controls has been used to prioritize effective and sustainable control solutions.^98^ The hierarchy of controls has been adapted to reflect TWH principles and can serve as a framework for addressing work-related psychosocial hazards.^98^ NIOSH recommends applying the five levels of the hierarchy in the following order:

1. eliminate negative working conditions and barriers to safety, health, and well-being;
2. substitute safer and healthier workplace policies, work processes, and practices;
3. redesign the work environment to enhance working conditions and improve safety, health, and well-being;
4. educate all employees and provide resources for improved knowledge; and
5. encourage or reinforce adoption of safe and healthy practices.

Workplace health and well-being interventions can also be conceptualized as another hierarchy: primary (prevention and mitigation of risk), secondary (treatment or early intervention following exposure), and tertiary (limiting further harms and rehabilitation to resume work).^135,136^

There are multiple approaches employers can take to mitigate work-related psychosocial hazards. These approaches can target the individual (e.g., health promotion and stress management programs) or the organization (e.g., work redesign) and can be delivered at primary, secondary, or tertiary prevention levels (Table 4).^136^ The general approach should start with applying primary prevention approaches at the broadest levels. In the case of psychosocial hazards, this means interventions that alter the work environment, rather than individually focused psychosocial supports.^1,138^ Organizational-level solutions approaches are likely to be more efficient, have a broader impact, and be more sustainable. In addition, primary prevention efforts benefit all workers, including those unable to access individual services. For these reasons, organizational interventions are the key recommended approach for improving psychosocial working conditions in various countries.^97,124,139^

Next, we describe the evidence for both organizational and individually focused interventions that address work-related psychosocial hazards. It is suggested that comprehensive approaches, which include both organizational and individual-level interventions, may be the most impactful and sustainable.^97,124,140^

### 5.2 | Effectiveness of organizational interventions

Aust et al. (2023) conducted a meta-review of 957 studies and found strong quality evidence for the effectiveness of organizational level interventions focusing on “changes in working time arrangements” and moderate evidence for “influence on work tasks or work organization,” “healthcare approach changes,” and “improvements of the psychosocial work environment.”^141^ They also found strong quality evidence for interventions about “burnout (chronic or long-lasting exhaustion related to work)” and moderate quality evidence for “various health and well-being outcomes.” The meta-review concluded that while organization-level interventions are still relatively rare, there is growing evidence that they, especially when combined with individual-level interventions, can be effective in promoting positive, healthy work.^142,143^ For further information on the effectiveness of organizational interventions, see Supporting Information S1: SI 6.

### 5.3 | Effectiveness of individual interventions

Many recent approaches that address work-related psychosocial hazards engage workers in various health promotion strategies. The rapid growth of workplace health promotion (WHP) programs related to stress and mental health conditions is indicative of this tendency to focus on individual approaches to managing psychosocial hazards. A study of 17,469 employed US adults from the 2015 National Health Interview Survey found that 46.6% reported at least one WHP practice was available at their workplace, and among those, 57.8% participated.^144^ A common feature of WHP is engagement in physical activity, and reviews indicate these interventions show promising results in reduced absenteeism and presenteeism.^145^ Individual interventions may also be easier to implement than organizational interventions.^136^

While there is a significant body of literature to support the effectiveness of individual approaches to managing psychosocial hazards, some qualifications to this observation should be noted.^146^ First, a review of stress management interventions found little research comparing the effectiveness of stress management interventions at the individual and organizational levels.^146,147^ Further reviews of individual-level interventions have also noted that effects can be short-lived or that data on long-term effects are absent altogether.^148,149^

### 5.4 | What is the most effective approach?

There is growing evidence to support a comprehensive approach in which integrated systems are developed that address all three elements of prevention (primary, secondary, and tertiary) for work-related psychosocial hazards (Table 4).^6,123,124,150^ That is, the more comprehensive an intervention may be, the greater the potential for impact.^151^

Also, a recent meta-analysis confirms that workplace resources applied at the individual, group, leaders, and organization levels are each related to employee well-being and performance.^123,150^ Other scholars have suggested that “approaches to workplace well-being interventions that selectively cross-fertilize and adapt elements of health promotion interventions offer promise for realizing a broader change agenda and for building inherently healthy workplaces.”^135^ Another recent systematic review has identified the most effective approaches, including both organizationally-and individually-focused approaches, as well as both primary and secondary approaches.^152^ In this systematic review, there was a promising number of interventions designed to incorporate both primary and secondary prevention methods, with 32% of interventions employing hybrid designs.

## 6 | SOCIETAL ACTIONS TO ADDRESS WORK-RELATED PSYCHOSOCIAL HAZARDS

As illustrated in this paper, exposure to work-related psychosocial hazards and associated mental health outcomes on workers, employers, and society is growing and creating an *urgent* need for action. Six actions are recommended: (1) increase awareness of this critical issue through a comprehensive public campaign; (2) increase etiologic, intervention, and implementation research; (3) initiate or augment surveillance efforts (to better capture incidence, prevalence, and costs of psychosocial hazards and their adverse effects); (4) increase translation of research findings into guidance for employers and workers; (5) increase the number and diversity of professionals skilled in preventing and addressing psychosocial hazards; and (6) develop a national regulatory or consensus standard to prevent and control work-related psychosocial hazards.

### 6.1 | Increase awareness of this critical issue through a comprehensive public campaign

The extent, severity, and burden of psychosocial hazards on workers, while known and addressed by some employers, is not acknowledged or acted upon by others.^82^ For prevention and control of work-related psychosocial hazards to be prioritized, awareness needs to be improved. Preventing them must become part of the organizational culture, similar to the way businesses acknowledge traumatic injuries or chemical hazards. To influence the culture, a broad-based campaign led by a coalition of business, labor, insurers, government agencies, and professional associations should be developed. The campaign should popularize the burden of work-related psychosocial hazards, the means to address them, and models of successful efforts. One recent step toward increasing awareness is the report by the Surgeon General on workplace mental health and well-being.^2^

### 6.2 | Increase etiologic, intervention, and implementation research

While there is a rich body of research on work-related psychosocial hazards and their adverse health effects, there are still knowledge gaps on their etiology, interventions, and implementation.^11,43,153^ There is a rather consistent body of research that certain psychosocial working conditions (job strain, effort-reward imbalance, job insecurity, and long work hours) are strongly linked with adverse health effects. There is still a need, however, for a greater understanding of causality.^154^ The evaluation by Madsen and Rugulies (2021) shows modest pooled relative risks less than 2.0, so residual confounding (a problem for observational studies with low relative risks) could be an issue.^154^ Moreover, most studies include self-reported data and could therefore be affected by differential bias.^154^ Further work, using job exposure matrices, will help mitigate limitations of self-reports of job demand and job control.

More intervention studies on the control of psychosocial hazards are also needed. Evaluation of workplace interventions that improve mental health is complex and requires sophisticated evaluation designs”^154^ “Future research should use mixed methods to evaluate organizational interventions by addressing how different mechanisms in specific contexts produce specific outcomes.”^153^ Research is also needed on how risk assessments can be utilized to study psychosocial hazards.^142^ While risk matrix approaches have been applied to other work-related hazards (e.g., nanoparticles, physical hazards), there is a need to evaluate risk matrix approaches’ utility and cost-effectiveness for exposure to psychosocial hazards.^142^

### 6.3 | Initiate or augment surveillance efforts

The need for national surveillance of work-related psychosocial hazards was recommended in a review of surveillance systems for psychosocial risks in 20 countries.^155^ The USA currently has limited surveillance of psychosocial hazards. Research and intervention priorities are driven by the extent to which the exposures and effects can be surveilled and addressed nationally. There is also a need for improved monitoring at the organizational level to drive prevention and control programs for psychosocial hazards.

At the organizational level, important surveillance efforts are the assessment of the workers’ and employers’ attitudes toward organizational practices.^140^ There are existing tools such as the 2021 NIOSH Worker Well-Being Questionnaire, the Harvard “Thriving” questionnaire, the NIOSH Quality of Worklife questionnaire, and others that can assess workers’ concerns.^156,157^ Additionally, questions about work-related psychosocial hazards have been added to periodic occupational supplements to the National Health Interview Survey to assess population-based prevalence (https://www.cdc.gov/niosh/topics/nhis/default.html). The RAND Corporation has also sponsored an American Working Conditions Survey (https://www.rand.org/pubs/research_briefs/RB9973-1.html). Data on work-related psychosocial hazards may also be found, to a limited extent, in the CDC Behavioral Risk Factor Surveillance System.^158^ However, for more complete assessment of the prevalence and incidence of work-related psychosocial hazards and their adverse effects, national surveillance systems should be augmented.

### 6.4 | Increase translation of research findings into guidance for employers and workers

Many employers lack knowledge of their responsibility for, and how to control, work-related psychosocial hazards, despite an adequate scientific literature to draw upon.^82,123,124,140^ “The lack of knowledge may be due to a number of factors including that psychosocial hazards are not tangible or easily observable and workplace psychological safety is a relatively new concept for some employers.”^82^ Anger et al. (2015, 2019) found that there was a lack of dissemination and implementation of effective interventions.^152,159^ There is a need to translate and distill scientific information and make it available to employers and workers. Concerted actions are needed to get effective information to employers and increase the likelihood that they will use it.^160^

### 6.5 | Increase the number and diversity of professionals skilled in preventing and addressing psychosocial hazards

There is a lack of mental health literacy nationally and a shortage of professionals who are knowledgeable about work-related psychosocial hazards. There are calls for training psychologists and occupational health professionals so that there are more professionals in occupational health psychology (OHP), but the response in terms of training new investigators and practitioners has not been sufficient.^161^ There is a need for more emphasis by government agencies, universities, professional associations, employers, and unions to increase the investment in training occupational health psychologists. There is also a need to bridge OHP and occupational safety and health to support a more central role for OHP in the OSH field.^161^ Additionally, it is useful to expand the knowledge base of OSH. For example, the Australian Institute of Health and Safety has developed a core OSH body of knowledge on psychosocial hazards for generalist OSH practitioners.^117^ Also, the role of Employee Assistance Programs (EAP) needs to be expanded and modernized to make them more impactful, including having EAPs provide both individually focused services and organizational-level interventions.^162^

### 6.6 | Develop a national regulatory or consensus standard to prevent and control work-related psychosocial hazards

The OSH Act of 1970 and the Federal Coal Mine Safety and Health Act of 1969 address the OSH of US workers. These standards were promulgated by the Mine Safety and Health Administration and the Occupational Safety and Health Administration (OSHA) generally with input from NIOSH and others through criteria documents, research, and testimony. Psychosocial hazards and effects are mentioned in the OSH Act as “psychological factors” but with limited specifications or emphasis.^163^

In developing a standard for work-related psychosocial hazards, it is useful to consider whether addressing these hazards would be best served by following the past approach for standards (e.g., a “specification” approach) or whether something different, such as a “performance” approach should be considered.^164^ The variations in businesses and the subjective nature of some psychosocial hazards and adverse effects may not readily lend themselves to the type of standards developed for chemical and physical hazards. Rather, a more general, performance-focused process for work-related psychosocial hazards may be more appropriate.

Another issue is whether a standard should be legally binding or voluntary (Table 5).^168,169^ The European Union has various work-related, psychosocial standards and laws, some of which have been implemented for more than 10 years.^168,169^ A voluntary workplace psychosocial standard has been in place in Canada since 2013.^102^ In 2021, the International Organization for Standardization also published a voluntary global standard, which is being utilized by various organizations.^103^ The American National Standards Institute has adopted the ISO standard as a Nationally Adopted International Standard.^103^ The USA could benefit by being consistent with the global effort to address work-related psychosocial hazards by developing a US-initiated standard.^1^

## 7 | CONCLUSIONS

There is compelling evidence that workers are increasingly being exposed to work-related psychosocial hazards resulting in harmful health and economic effects to them, their companies, their communities, and to nations. Action needs to be taken to reverse this trend.^83^ In this paper, evidence for these hazards is reviewed, and six remedial actions that may ameliorate a growing and significant public health problem are presented. When done comprehensively, preventing and addressing work-related psychosocial hazards will help protect workers and promote work as a means to achieving greater health and well-being for all.

## Supplementary Material

Supporting Information

## Figures and Tables

**TABLE 1:** Psychosocial aspects of work and related hazards.

Psychosocial aspects of work	Associated psychosocial hazards
Job content	Lack of variety or short work cycles; fragmented or meaningless work; under-use of skills; high uncertainty; continuous exposure to difficult clients, patients, pupils, etc.
Workload and work pace	Work overload or too little work, machine pacing, high levels of time pressure, continually subject to tight deadlines
Work schedule	Shift work, night shifts, inflexible work schedules, unpredictable hours, long or unsociable hours
Control	Low participation in decision-making; lack of control over workload, pacing, shift working, etc.
Environment and equipment	Inadequate equipment availability, suitability, or maintenance; poor environmental conditions such as lack of space, poor lighting, excessive noise
Organizational culture and function	Poor communication; low levels of support for problem solving and personal development; poor managerial support; lack of definition of, or agreement on, organizational objectives
Interpersonal relationships at work	Social or physical isolation, poor relationships with superiors, interpersonal conflict, lack of social support, harassment, bullying, poor leadership style, third-party violence
Role in organization	Role ambiguity, role conflict, responsibility for people
Career development	Career stagnation and uncertainty, under-promotion or over-promotion, poor pay, job insecurity, low social value of work
Home-work interface	Conflicting demands of work and home, low support at home, problems relating to both partners being in the labor force (dual career)

*Source:* Adapted from Mellor et al. (2011)^169^, Leka and Jain (2014),^168^ and Cox et al. (2005)^21^.

**TABLE 2:** Selected scientific literature describing the association between occupation, psychosocial hazards, and adverse behavioral, mental health, and physical effects.

Effects	Representative references
Absenteeism	Dobson et al., 2020^15^; Sitarević et al., 2023^16^
Accidents	EU-OSHA, 2007^17^; Gomez-Ortiz et al., 2018^18^
Alcohol and drug use	Richter et al., 2021^19^; Virtanen et al., 2015^20^
Anxiety	Cox et al., 2005^21^; Niedhammer et al., 2021^11^; Harvey et al., 2017^22^
Behavioral disorders	Chamoux et al., 2018^23^; Harvey et al., 2017^22^
Burnout	Maslach and Leiter, 2016^24^; Schaufeli et al., 2009^25^; Ahola et al., 2007^26^; Kivimäki et al., 2012^27^; O’Connor et al., 2018^28^
Cardiovascular disease	Niedhammer et al., 2021^11^; Pega et al., 2021^29^; Kivimäki et al., 2006^30^; Belkic et al., 2004^31^; Kuper et al., 2002^32^; Schnall et al., 1998^33^
Cigarette smoking	Conway et al., 1981^34^; van den Berge et al., 2021^35^
Cognitive impairment	Grzywacz et al., 2016^36^; Elovainio et al., 2009^37^; Peterson et al., 2008^38^
Depression	Niedhammer et al., 2021^11^; Theorell et al., 2015^39^; Leka, 2010; Cox et al., 2005^21^; Mikkelson et al., 2021^40^; WHO, 2022^41^; Rugulies et al., 2023^42^; Madsen et al., 2017^43^; Rugulies et al., 2017^44^
Fatigue	Åkerstedt et al., 2004^45^; Tang et al., 2016^46^; Jalilian et al., 2019^47^
Health-related quality of life (HRQL)	Ray et al., 2021^48^; Bhattacharya and Ray, 2021^49^; Ray et al., 2014^50^
High blood pressure	Schnall et al., 1998^33^; Rosenthal and Alter, 2012^51^
Migraine headache	Wilkins and Beaudet, 1998^52^; Urhammer et al., 2020^53^; Magnavita, 2022^54^
Mood disorders	Lovelock, 2019^6^; Netterstrøm et al., 2008^55^; Woo and Postolache, 2008^56^
Negative emotional reactions	Jordan et al., 2002^57^; West et al., 2016^58^
Obesity	Ostry et al., 2006^59^; Kivimäki et al., 2003^60^; van den Berge et al., 2021^35^
Poor self-reported health	Stadin et al., 2019^61^; Niedhammer et al., 2022^62^
Posttraumatic stress disorder	Spence Laschinger and Nosko, 2015^63^; Nielsen et al., 2015^64^; Rudkjoebing et al., 2020^65^
Sickness Absence	Kivimäki et al., 2003^60^; Duchaine et al. 2020^66^; Goorts et al., 2020^67^
Sleep disturbance	Rugulies et al., 2009^68^; Peterson et al., 2008^38^; Åkerstedt, 1995^69^; Rudkjoebing et al., 2020^65^
Stress reaction	Nieuwenhuijsen et al., 2010^70^; WHO 2003^71^; van der Molen et al., 2020^72^
Subjective well-being decrease	Ray, 2021^73^; de Jonge et al., 2000^74^
Suicide and suicidal ideation	Niedhammer et al., 2021^11^; Woo and Postolache, 2008^56^; Milner et al., 2018^75^; Aronsson et al., 2017^76^
Work/family imbalance	Hämmig et al., 2011^77^; Jerg-Bretzke et al., 2020^78^

**TABLE 3:** Estimated prevalence of work-related psychosocial hazards in the United States in 2018 based on the Quality of Work Life supplement to the General Social Survey.

Psychosocial hazard	Specific job characteristics	Prevalence in percentage (%)
Job content	Stressful work	29
	Does not allow to apply skills	7
	Do not learn new things	7
	Face conflicting demands	23
	Requires repeated heavy lifting	51
Workload and work pace		
	Not enough time to get job done	16
	Not enough people to get job done	25
	Requires to work very fast	69
	Job demand interferes family life	43
Work schedule		
	Inability to take time off when needed	26
	Doing irregular or rotating shifts	13
Control		
	Do not take part in decision-making	24
Environment and equipment		
	Lack of smoothness in the running of workplace	21
	Poor safety and health conditions	5
Role in organization		
	Does not have enough information to do the job properly	5
	Do not know what is expected at work	5
Career development		
	Job insecurity	10
	Earnings not fair compared to other workplaces	37
	Little chances of promotion	40
Interpersonal relationships at work		
	No trust in management	17
	Supervisor not helpful enough	12
	Not treated with respect	6
	Discriminated for	
	Age	8
	Sex	6
	Race	6
	Harassed at work	
	Sex	3
	Other	7

*Note:* Obtained from General Social Survey (GSS), 2018–NIOSH Quality of Work Life (QWL) supplement. The sample data (*N* = 1413) is weighted to represent the US working population. The weights (WTSALL) are provided by the GSS to account for the probability of selection, subsampling and number of adults in the household. This helps to address the subsampling of certain demographic and geographical groups. To know more, consult the GSS Codebook (https://gssdataexplorer.norc.org/gssweighting).

**TABLE 4:** Model for categorizing workplace stress management preventive interventions.^a,b^

Level	Primary prevention^a^	Secondary prevention^a^	Tertiary prevention^a^	Outcome measures^c^
Organizational	Improving work content, fitness programs, ^b^ career development	Improving communication and decision-making, conflict management, fitness programs^b^	Vocational rehabilitation, outplacement	Productivity, turnover, absenteeism, financial claims
Individual and organizational interface	Time management, improving interpersonal skills, work/home balance	Peer support groups, coaching, career planning	Posttraumatic stress assistance programs, group psychotherapy	Job stressors such as demands, control, support, role ambiguity, relationships, change, burnout
Individual	Pre-placement medical examination, didactic stress management	Cognitive behavioral techniques, relaxation	Rehabilitation after sick leave, disability management, case management, individual psychotherapy	Mood states, psychosomatic complaints, subjective experienced stress, physiological parameters, sleep disturbances, health behaviors

*Source:* Adapted from De Jonge and Dollard (2002)^137^ and Dinos et al. (2017).^136^

a Primary prevention involves interventions to prevent causal factors of stress-related symptoms at work. Secondary prevention involves interventions to reduce the severity or duration of stress-related symptoms. Tertiary prevention involves interventions to provide rehabilitation and maximize functioning among those with chronic stress-related or health conditions impacting work.^136^

b Fitness programs could be a primary prevention strategy if they promote or maintain health to protect workers while doing their jobs. They could, however, also be a secondary prevention strategy, for example, after illness or injury. Also fitness programs could be characterized as health promotion programs. Having policies to support health promotion (e.g., providing opportunities to participate during work hours) would be an organizational-level intervention, while the program components themselves (e.g., employees using onsite exercise facilities; attending seminars) are more individual-level intervention approaches.

c These are level-specific outcomes. It would be possible to measure intervention outcomes across levels. The outcomes shown in the table are just the most prominent examples of outcomes associated with different intervention levels/approaches.

**TABLE 5:** Examples of existing standards to address work-related psychosocial hazards.

Name of standard	Place, date promulgated	Description
Guidance on the management of psychosocial risks in the workplace (Leka et al., 2011)^105^	United Kingdom, 2011	Voluntary - provides guidance and good practice on assessing and managing psychosocial risks at work.
National standard for Canada for psychosocial health and safety in the workplace (Can/CSA, 2013)^102^	Canada, 2013	Voluntary - focused on promoting workers’ psychological health and preventing psychological harm due to work-related factors.
Stress Check Program (Kawakami & Tsutsumi, 2016)^165^	Japan 2015	Mandatory national policy for monitoring and screening psychological stress in the workplace.
ISO 45003: Occupational health and safety at work - guidelines for managing psychosocial risks (ISO, 2021)^103^	International, 2021	Voluntary consensus standard; Guidance on the management of psychosocial risks and promoting well-being at work.
Managing psychosocial hazards at work: code of practice (Work Health and Safety Commission, 2022)^166^	Australia, 2022	Mandatory - code is intended to provide some practical guidance on how to comply with general language in the legal, standard imposed by law.

*Note:* See Cobb (2022)^1^, Jain et al. (2021),^167^ and Lovelock (2019)^6^ for a broader assessment of international regulations and guidance.

## Data Availability

Data sharing is not applicable to this article as no datasets were generated or analyzed during the current study.
