# Supplementary material for: An urgent call to address work-related psychosocial hazards and improve worker well-being
Source: Am J Ind Med. Author manuscript; Available in PMC 2025 Apr 9. (PMC11980372; doi:10.1002/ajim.23583)
Supplement: Supporting Information [file NIHMS2062032-supplement-Supporting_Information.docx]

# Supporting Information (SI)

## SI 1. Worker mental health and well-being in the workplace

Research indicates that mental health is worsening in the U.S. population, including working adults.^1,2,3,4,5^ A large cohort study that utilized National Health Interview Study (NHIS) data found that among working-age adults the prevalence of psychological distress increased 40% from 1999 to 2018.^1^ Construction workers are one of the occupational groups with high rates of suicide.^6^

The impact of mental disorders on society is substantial.^7-9^ Mental disorders have large direct costs as well as many indirect costs related to the workplace: “… increased rate of short-term disability, safety incidents, absenteeism and presenteeism, underperformance and unrealized output, stress imposed on team members, overtime and overstaffing to cover sick-day absence, and hiring correlated to recruitment and retention.”^10^ Furthermore, mental health conditions are generally more costly and of longer duration than physical health ones.^8,9^

More broadly, worker mental health is a component of worker well-being. Worker well-being, adopted from Chari et al. (2022) is: “an integrative concept that characterizes quality of life with respect to an experience of positive perceptions and the presence of constructive conditions at work and beyond that enables workers to thrive and achieve their full potential.”^11^ The importance of job quality in determining the overall well-being of a worker is highlighted in a recent study. Utilizing Gallup-ShareCare Well-Being index data, the study shows that, controlling for covariates and other nonwork-related contributors to wellbeing, job satisfaction accounts for a 14% increase in life evaluation (satisfaction) scores.^12^ Acknowledging that work is a principal contributor to worker well-being, it is imperative to consider the role of work design, specifically psychosocial hazards, as a determinant of health. Moreover, as noted by the U.S. Surgeon General in a recent report, the workplace should play a foundational role in promoting mental health and well-being of workers and communities.^13^

## **SI 2.** Exposure to work-related psychosocial hazards

Several surveys have investigated the prevalence of exposure to psychological hazards in the U.S. The NIOSH Quality of Work Life (QWL) survey is administered every four years as part of the General Social Survey (GSS), a biannual cross-sectional survey of U.S. households that yields a nationally representative sample of the civilian, non-institutionalized, English-speaking U.S. adult population. Using 2002-2014 QWL data, Ray et al. (2017) found a 29% prevalence of overwork; 66% of fast work; 13% not being given freedom to decide own work; 15% lack of supervisor support; 9% that could not rely on coworkers; 14% with no opportunity to learn; 14% with low job security; 29% with poor fringe benefits; 7% with poor safety and health conditions; 43% with poor work-family balance; 31% with work stress; and 10% not satisfied with their job.^14^ Using the same data source, Myers et al. (2019), found a statistically significant increase in job strain (e.g., low job control) and work-family conflict between 2002 and 2014.^15^

Work-related exposure to psychosocial hazards also can be seen in data from the 2016 National Study of Employers (NSE).^16^ The NSE is a U.S. survey representing 920 employers with 50 or more employees in various industries. “Although there are similar surveys by employer membership organizations, consulting firms, and government agencies, the NSE is notable in that it is the only survey of employers in the U.S. that comprehensively assesses a broad array of programs, policies, and benefits designed to address the changing needs of employees among a nationally representative group of employers.”^16^ While the survey showed many changing policies and practices favorable to workers between 2008 and 2016 and determined the extent (percentages) to which “at least some workers” benefited from the policies, there were striking findings. When the responses were categorized by businesses where “almost all employees” were considered: 41% had no control over when to take breaks; 90% had no control of which shift to work; 48% were not allowed a gradual return after childbirth or adoption; and 53% were not allowed to take time off during the workday to attend to important family or personal needs without loss of pay.^16^

## SI 3. Background mental health disorders

From 2011 to 2020, a total of 29,870 (0.32%) of injuries/illness with days away from work were identified as having a primary nature of “Mental Disorders and Syndromes” in the BLS Survey of Occupational Injuries and Illnesses (SOII).^17^ This varied by state, and CA had the highest number (14,520) and proportion (1.2%) of MH cases. Similar MH claim proportions and differences between states have been found in WC systems using basic coded data.

One of the most common mental health disorders is major depressive disorder (MDD). MDD can severely affect individuals’ daily functioning and is the leading cause of disability worldwide.^18^ In 2018, 6.3% of employed U.S. adults had MDD.^19^ Anxiety disorders are another common mental health condition that can affect workers. People with anxiety disorders find it difficult to control their worry, which may cause impairment in functioning in their work. In 2019, one in six (15.6%) U.S. adults experienced anxiety symptoms that were either mild (9.5%) or moderate (3.4%).^20^

Depression and anxiety are highly comorbid. Over half (57.5%) of individuals with depression also have an anxiety disorder.^21^ It is important to note that many of the statistics presented here on depression and anxiety in the U.S. are based on the general population and include both employed and unemployed individuals. Unemployed individuals are twice as likely to have been diagnosed with depression as employed individuals.^22^

Another category of mental health conditions is substance misuse disorders. Alcohol and other substance use and their related impacts on employment, safety, and well-being are significant public health challenges. Among employed adults, 8.7% have current alcohol or illicit drug use disorders, and 8.5% report that they are in recovery or have recovered from a substance use problem.^23^

## SI 4. Economic burden of psychosocial hazards

Greenberg et al. (2021) estimated the economic burden of adults with MDD in the United States in 2010 and 2018 using a framework for evaluating the incremental economic burden of adults with MDD compared to adults without MDD and including burden of comorbid conditions suffered by these groups.^19^ They used a medical costs and productivity losses approach and concluded that the number of adults with MDD increased from 15.5 to 17.5 million in the period examined. The incremental economic burden of adults with MDD increased by 37.9% from $236.6 billion to $326.2 billion in 2020 values. These estimates included productivity related costs due to presenteeism, not just absenteeism, and suicide-related costs. The study concluded that absenteeism and presenteeism costs (that they named “workplace costs”) “… accounted for the largest portion of the growing economic burden of MDD as this population trended younger and was increasingly likely to be employed.”^19^ The estimates presented by Greenberg and colleagues, however, do not address the causal link between work-related hazards and economic burden. However, illness estimates that provide a clear attribution or contribution of work to MDD costs are not generally available in the published literature. Even though these costs are not fully attributed to work, they represent a true burden for workers, employers, and society overall. Trautmann et al. (2016) estimated the worldwide economic burden of mental disorders among the general population using three prevalence-based methods (human capital method, value of a statistical life method, and macroeconomic approach). In this analysis, the economic burden for mental disorders was estimated to be $6.1 to $16.3 trillion by 2030.^24^

## SI 5. Costs of workers’ compensation claims

Based on 2015-2017 data from the National Council on Compensation Insurance (NCCI), which collects population data for carrier-insured employers from 38 states, the mean costs of a lost-time mental health disorder and mental stress claim were $59,418 and $35,151 respectively. This is less than the mean cost ($60,956) for all other injury/illness types. In contrast, other studies have suggested that physical-mental claims tend to be more costly than WC claims with just physical diagnoses.^25^

## **SI 6. Effectiveness of organizational interventions**

A review of 83 studies, that distilled intervention approaches into four categories (flexible work and scheduling; job and task modifications; relational and team dynamic initiatives; and participatory process interventions) found strategies aiming to change work conditions have the potential to improve worker well-being.^26^ These researchers concluded that regardless of type, interventions involving increased control and opportunities for worker participation more reliably improve worker well-being, suggesting these components are critical elements of successful interventions.

Another review of healthy workplace practices examined five intervention categories: work-life balance, employee growth and development, health and safety, recognition, and employee involvement.^27^ This review concluded that the link between these practices and employee and organizational outcomes is contingent on effective organizational communication and alignment of workplace practices within an organizational context. In other words, it is important to consider organizational context when developing intervention programs.

Published information suggests that leadership training, that is, training supervisors and managers, can be an important intervention strategy. A systematic review of 29 randomized controlled trials, published between 2000 and 2020, found that leadership training was quite effective in impacting worker stress and well-being of supervised employees.^28^ However, one systematic review found that 3 of the 4 studies with the highest level of evidence failed to achieve a positive outcome.^187^ These studies mainly used training interventions to target outcomes such as job satisfaction,^188^ work engagement, social support at work, and need for recovery.^29,30^

## Supporting Information References

1. Daly M. Prevalence of psychological distress among working‐age adults in the United States, 1999–2018. *Am J Public Health.* 2022;*112*(7):1045‐1049.
2. MacMillan A. *4 possible reasons why mental health is getting worse.* 2023. <https://www.health.com/condition/depression/8-million-americans-psychological-distress>. Accessed 6 March 2024.
3. Reinert M, Fritze D, Nguyen, T. *The state of mental health in America 2022.* 2021. <https://mhanational.org/sites/default/files/2022%20State%20of%20Mental%20Health%20in%20America.pdf> Accessed 6 March 2024.
4. Han B, Crosby AE, Ortega LAG, Parks SE, Compton WM, Gfroerer J. Suicidal ideation, suicide attempt, and occupations among employed adults aged 18–64 years in the United States. *Compr Psychiatry.* 2016; *66*:176-186. <https://doi.org/10.1016/j.comppsych.2016.02.001>
5. Hedegaard H, Curtin SC, Warner M. Increase in Suicide Mortality in the United States, 1999–2018. *NCHS Data Brief.* US Department of Health and Human Services, Centers for Disease Control and Prevention, National Center for Health Statistics, 2020. <https://stacks.cdc.gov/view/cdc/86670>
6. Dong XS, Brooks RD, Brown S, Harris W. Psychological distress and suicidal ideation among male construction workers in the United States. *Am J Ind Med.* 2022; *65*(5):395-408. <https://doi.org/10.1002/ajim.23340>
7. Arias D, Saxena S, Vergent S. Quantifying the global burden of mental disorders and their economic value. *eClinical Medicine*; 2022; *54:*101675. <https://doi.org/10.1016/j.eclinm.2022.101675>
8. Bloom DE, Cafiero E, Jane-Llopis E, et al. *The Global Economic Burden of Non-Communicable Diseases.* 2011; <https://www3.weforum.org/docs/WEF_Harvard_HE_GlobalEconomicBurdenNonCommunicableDiseases_2011.pdf>. Accessed 6 March 2024.
9. Roehrig C. Mental disorders top the list of the most costly conditions in the United States: $201 billion. *Health Aff.* 2016; *35*(6):1130-1135. <https://doi.org/10.1377/hlthaff.2015.1659>
10. Goetzel RZ , Roemer EC , Holingue C , et al. Mental health in the workplace: a call to action. Proceedings from the mental health in the workplace: Public health summit. *J Occup Environ Med*. 2018;*60*(4):322‐330. <https://doi.org/10.1097/JOM.0000000000001271>
11. Chari R , Sauter SL , Petrun Sayers EL , Huang W , Fisher GG , Chang C‐C . Development of the National Institute for Occupational Safety and Health worker well‐being questionnaire. *J Occup Environ Med*. 2022; *64*(8):707‐717. <https://doi.org/10.1097/JOM.0000000000002585>
12. Ray TK . Work related well‐being is associated with individual subjective well‐being. *Ind Health*. 2021;*60*(3):242‐252. <https://doi.org/10.2486/indhealth.2021-0122>
13. DHHS. The US Surgeon General's framework for workplace mental health & well‐being. US Dept of Health and Human Services, Office of Surgeon General. 2022. <https://wellnessatnih.ors.od.nih.gov/news/Pages/The-U.S.-Surgeon-General%E2%80%99s-Framework-for-Workplace-Mental-Health-and-Well-Being.aspx> Accessed 6 March 2024.
14. Ray TK, Kenigsberg TA, Pana-Cryan R. Employment arrangement, job stress, and health-related quality of life. *Saf Sci.* 2017; *100*:46-56. <https://doi.org/10.1016/j.ssci.2017.05.003>
15. Myers S , Govindarajulu U , Joseph M , Landsbergis P . Changes in work characteristics over 12 years: findings from the 2002–2014 US National NIOSH Quality of Work Life Surveys. *Am J Ind Med.* 2019;*62*(6):511‐522. <https://doi.org/10.1002/ajim.22971>
16. Matos K , Galinsky E , Bond JT . National Study of Employers. 2016. Society for Human Resource Management. <https://cdn.sanity.io/files/ow8usu72/production/d73a7246cc3a3fef4ad2ece1e3d5aa4eaec2f263.pdf>. Accessed 6 March 2024.
17. BLS. Survey of Occupational Illnesses and Injuries (SOII). Table R67. *US Dept of Labor, Bureau of Labor Statistics,* 2023. <https://www.bls.gov/iif/nonfatal-injuries-and-illnesses-tables.htm>
18. 179 Friedrich MJ. Depression is the leading cause of disability around the world. *JAMA*. 2017; *317*(15):1517. <https://doi.org/10.1001/jama.2017.3826>
19. Greenberg PE, Fournier A-A, Sisitsky T, et al. The economic burden of adults with major depressive disorder in the United States (2010 and 2018). *Pharmacoeconomics.* 2021: *39*(6):653-665. <https://doi.org/10.1007/s40273-021-01019-4>
20. Terilzzi EP, Villaroel MA. Symptoms of generalized anxiety disorder among adults: United States, 2019. *US Dept of Health and Human Services, Centers for Disease Control and Prevention, National Center for Health Statistics,* 2020. <https://www.cdc.gov/nchs/products/databriefs/db378.htm>. Accessed 6 March 2024.
21. Kessler RC , Angermeyer M , Anthony JC , et al. Lifetime prevalence and age‐of‐onset distributions of mental disorders in the World Health Organization's World Mental Health Survey Initiative. *World Psychiatry: off J World Psychiatric Assoc (WPA)*. 2007; *6*(3):168‐176. <https://doi.org/10.1001/archpsyc.62.6.593>
22. Harnois G, Gabriel P. Mental health and work: Impact, issues, and good practices. *International Labour Organization,* 2000. <https://www.ilo.org/skills/pubs/WCMS_108152/lang--en/index.htm>. Accessed 6 March 2024.
23. Frone MR, Chosewood LC, Osborne JC, Howard JJ. Workplace supported recovery from substance use disorders: Defining the construct, developing a model, and proposing an agenda for future research. *Occup Health Sci.* 2022; *6*(4):475-511. <https://doi.org/10.1007/s41542-022-00123-x>.
24. Trautman S, Rehm J, Wittchen HU. The economic cost of mental disorders. *EMBO Rep.* 2016. *17*(9):1245-1249. <https://doi.org/10.15252/embr.201642951>
25. Spidell B . Examining PTSD‐What's the impact on future workers' compensation costs. *National Council on Compensation Insurance.* <https://www.ncci.com/Articles/Pages/Insights-Examining-PTSD-Impact-on-Future-WorkersComp-Costs.aspx> Accessed 6 March 2024.
26. Fox KE , Johnson ST , Berkman LF , et al. Organisational‐ and group‐level workplace interventions and their effect on multiple domains of worker well‐being: a systematic review. *Work Stress*. 2022; *36*(1):30‐59. <https://doi.org/10.1080/02678373.2021.1969476>
27. Grawitch MJ, Gottschalk M, Munz DC. The path to a healthy workplace: A critical review linking healthy workplace practices, employee well-being, and organizational improvements. *Consul Psychol J Prac Res.* 2006; *58*(3):129-147. <https://doi.org/10.1037/1065-9293.58.3.129>
28. Hammer LB, Allen SJ, Leslie JJ. Occupational stress and well-being: Workplace interventions involving managers/supervisors. In Lapierre L, Cooper C, (eds). *Cambridge Companion to Organisational Stress and Well-Being.* Cambridge University Press; 2023.
29. Lamontagne AD , Keegel T , Louie AM , Ostry A , Landsbergis PA. A systematic review of the job‐stress intervention evaluation literature, 1990–2005. *Int J Occup Environ Health.* 2007;*13(*3):268‐280. <https://doi.org/10.1179/oeh.2007.13.3.268>
30. Oude Hengel KM, Blatter BM, Joling CI, van der Beek AJ, Bongers PM. Effectiveness of an intervention at construction worksites on work engagement, social support, physical workload, and need for recovery: Results from a cluster randomized controlled trial. *BM Public Health.* 2012; *12*(1):1008. <https://doi.org/10.1186/1471-2458-12-1008>
